# Editorial: African Swine Fever

**DOI:** 10.3389/fvets.2020.632292

**Published:** 2021-01-27

**Authors:** Jose Manuel Sánchez-Vizcaíno, Alberto Laddomada, Marta Martínez Avilés

**Affiliations:** ^1^VISAVET Health Surveillance Centre and Animal Health Department, Veterinary School, Complutense University of Madrid, Madrid, Spain; ^2^Advisor of the EU VACDIVA Project, Sardinia, Italy; ^3^Centro de Investigación en Sanidad Animal (INIA-CISA), Madrid, Spain

**Keywords:** ASF, vaccine, diagnosis, wild boar-domestic pig interface, epidemiology and control

The African swine fever (ASF) virus' third and deadliest tour outside Africa began in 2007, on a ship from the east coast of Africa bound for the port of Poti in Georgia (1). Once again, contamination of local pigs with food residues from the ship produced the first outbreak, which quickly spread among the pig and wild boar population from South to North and from East to West. ASF is currently present in four continents, affecting more than 50 countries, and causing millions of dead pigs.

More than seven different epidemiological scenarios are observed with different risk factors involved in each of them. This epidemiological situation has greatly changed the international pig market. The enormous losses of pig population affected Asia and mainly China (which represented 50% of the world pig population), in particular in its backyard and family population (50% of total Chinese production) which has been the worst affected by ASF (2). Due to this situation, China does not currently produce the necessary number of pigs to meet the country's needs, a situation that will continue for a while. This demand for pig meet has been supplied to date with important exports from the EU, USA, and Canada, and, to a lesser extent, Latin America (Brazil, Chile, and Mexico) (3). The recent infection of ASF in Germany and of Brazil with classical swine fever (CSF) (4) may change export flows and livestock movements, as well as the risk of ASF entry into other countries.

There are a few examples where ASF control has been possible despite infection in wild boar: Sardinia (Italy), affected since 1978 with a three-host epidemiological cycle in which free-ranging pigs played the most important role as virus source and reservoir, is now close to eradication (5). The last domestic pig outbreak in Sardinia was reported in September 2018 and the last finding of ASF virus in two wild boar carcasses was in April 2019 (6). Spain and Portugal were also able to achieve ASF eradication with a localized epidemiological scenario that included extensive pig production, ticks, and wild boar (7). On the other hand, the difficulty to eradicate the disease in other areas of Europe where the infection is very widespread and wild boar is the main virus host has been confirmed by the re-emergence of ASFV in Estonia last August (6), where no virus had been detected in the previous 18 months (8).

This Research Topic brings together 10 articles with updated knowledge on ASF pathology, diagnosis, vaccine development, epidemiology, and control and eradication.

ASF can cause different clinical courses, from peracute to chronic, depending on virus virulence, infective doses, or exposure route among others. Salguero reviews key clinical signs and lesions in domestic and wild pigs (Eurasian and African) infected with virus of different virulence. The acute form of the disease was observed in the first outbreak of ASF in Vietnam in 2019, as described by Nga et al., with high mortality and case fatality rate involving 3 farms. The first farm took longer to suspect ASF so clinical signs were observed for a month. Pig farming in Vietnam has low to no biosecurity measures to prevent the disease and feeding pigs with leftovers from cooking is common. CSF and PRRS are also present in Vietnam, both of which produce similar clinical signs to ASF, making the differential diagnosis more difficult. In this sense, the assay with high sensitivity and specificity developed and validated by Aira et al. to simultaneously detect ASF and CSF antibodies could help the timely recognition of ASF which is one of the most powerful tools to prevent its spread across huge geographic areas.

In effect, so far there have been no vaccines available to prevent or control ASF worldwide, and attempts to develop a safe vaccine have historically failed. Sang et al. review the key studies that have evaluated major approaches for the development of ASFV vaccines (live attenuated virus, inactivated virus, subunit vaccines, and live-vectored and DNA-based subunit vaccine candidates). The best vaccine candidates so far seem to be naturally attenuated viruses or produced by targeted gene deletions.

Despite the absence of vaccination or treatment, eradication has been possible in different epidemiological contexts. Risk factors together with the specific surveillance and intervention strategies to tailor them are reviewed by Danzetta et al. in each of the 11 countries that were able to eradicate this challenging disease, which in occasions has lasted for decades. The Italian island of Sardinia is one of the latest examples where, after more than 40 years of endemicity, is on its way to eradication after launching a risk-based plan adapted to the local situation which considered a three-host epidemiological cycle (wild boar, illegal free-ranging pigs, and domestic pigs) (Loi et al.). Frequent interactions between free-ranging pigs and wild boar populations and for long periods of time, particularly at water points, were observed with camera-traps by Cadenas-Fernández et al. in Sardinia.

Unfortunately, ASF in most of the European countries currently affected is nowhere near eradication. One of the challenges the European Union (EU) is facing is the high rate of infection in wild boar. Camera-traps were also used by Morelle et al. in a Polish National Park forest to assess the impact of ASF and hunting to control the disease in wild boar populations. Their study indicated that the intense hunting actions to control population during an acute ASF epidemic alone has a low additional impact on population decline compared with to the high mortality caused by the disease alone. Croft et al. reach a similar conclusion in their study about the hypothetical spatial and temporal patterns of ASF in wild boar following a hypothetical introduction of ASF in an abundant but low density wild boar population area of England. In isolated limited populations of wild boar, the model results show ASF fails to produce a self-sustaining disease. Finally, the epidemiological analyses of wild boar surveillance data by Martínez-Avilés et al. reveal that the expected increase in antibody detection is not always correlated with the time ASF has been present in an area, a potential explanation of which could be the circulation of less virulent strains in certain areas.

In summary, the articles in this Research Topic discuss important features of ASF and approaches to prevent and control it, which work best when adapted to the local situation, together with the latest developments and innovations in ASF research, advancing our understanding of this challenging disease. We trust readers will find these articles as stimulating to read as they were to edit.

## Author Contributions

JS-V wrote the introduction and final version of the editorial. MM wrote the articles' summaries and final remarks. AL amended and revised the final version. All authors contributed to the article and approved the submitted version.

## Conflict of Interest

The authors declare that the research was conducted in the absence of any commercial or financial relationships that could be construed as a potential conflict of interest.
